# Knowledge and clinical use of bioactive dental materials among a sample of general and paediatric dentists in Costa Rica: a cross-sectional study

**DOI:** 10.1038/s41405-026-00497-y

**Published:** 2026-11-08

**Authors:** Yanciny Ortega-Soto, Joseph Ulate-Jimenez, Adrian Gomez-Fernandez

**Affiliations:** Postgraduate Program in Paediatric Dentistry, Faculty of Dentistry, University of Costa Rica, San José, Costa Rica

**Keywords:** Dental biomaterials, Continuing professional development in dentistry

## Abstract

**Background:**

Understanding how dentists perceive and integrate bioactive dental materials into clinical practice is important to identify potential gaps between knowledge and use.

**Objective:**

To assess knowledge, conceptual understanding, and clinical use of bioactive dental materials among general dentists and paediatric dentists in Costa Rica.

**Methods:**

A cross-sectional survey was conducted among 240 licensed dentists (185 general dentists and 55 paediatric dentists) using a self-administered online questionnaire. The instrument assessed sociodemographic characteristics, familiarity with bioactivity concepts, recognition of bioactive materials, and patterns of clinical use. Topical fluoride agents were included to explore potential conceptual ambiguity. Descriptive and inferential analyses were performed using appropriate bivariate tests and multivariable logistic regression models.

**Results:**

Overall, 84.2% of participants reported familiarity with bioactivity. Paediatric dentists showed higher familiarity than general dentists (98.2% vs. 80.0%; *p* = 0.001), greater accuracy in identifying the correct definition (92.7% vs. 74.6%), and higher self-perceived knowledge (44.4% vs. 22.3%; *p* = 0.002). Bioactive materials were used by 65.3% of respondents, with paediatric dentists reporting a higher frequency of use (*p* = 0.027). After adjustment for age, sex, geographic practice location, and public-sector involvement, paediatric dentists had higher odds of correctly identifying the definition of a bioactive material (aOR = 4.98; 95% CI: 1.57–15.74; *p* = 0.006) and of using bioactive materials in at least 25% of clinical cases (aOR = 3.26; 95% CI: 1.38–7.69; *p* = 0.007).

**Conclusions:**

Knowledge and clinical use of bioactive dental materials differed between general dentists and paediatric dentists. Paediatric dentists demonstrated greater conceptual understanding and more frequent use, which may reflect differences in postgraduate training and clinical exposure.

## Introduction

Modern restorative dentistry has progressively shifted from conventional, bioinert materials toward biologically driven and minimally invasive approaches. Whereas traditional restorative materials primarily replace lost tooth structure, bioactive dental materials actively interact with dental tissues by releasing ions, promoting remineralization, modulating the oral environment, and supporting tissue repair through favourable biological responses [1, 2]. Glass ionomer cements, calcium silicate–based cements (e.g., mineral trioxide aggregate and Biodentine®), giomers, and functionalized resin-based materials exemplify restorative options with established or emerging bioactive properties [3, 4].

Bioactive materials are particularly relevant in paediatric dentistry, where preservation of pulp vitality, caries control, and reduction of operative intervention are central clinical priorities. Their use in pulp capping, pulpotomy procedures, and minimally invasive restorative techniques has demonstrated favourable biocompatibility and predictable clinical outcomes, consistent with contemporary preventive and regenerative paradigms [5, 6]. Paediatric dentists were selected as the specialist comparison group because bioactive materials have particular relevance to paediatric clinical practice, where vital pulp therapy, caries management, and minimally invasive restorative approaches are frequently employed. This specialty therefore provides a clinically relevant comparator for exploring whether postgraduate training and repeated exposure to these materials are associated with differences in knowledge and patterns of use relative to general dental practice.

Despite these advantages, the clinical adoption of bioactive materials remains heterogeneous worldwide, reflecting a persistent gap between scientific knowledge and real-world clinical implementation. Previous studies have reported marked variability in dentists’ knowledge, confidence, and frequency of use, influenced by undergraduate training, clinical experience, and access to continuing professional development [7, 8]. In addition, conceptual ambiguity surrounding the definition of bioactivity and its differentiation from conventional preventive strategies may further limit the rational integration of these materials into daily clinical practice [9].

Most available evidence on dentists’ perceptions and clinical adoption of bioactive materials originates from high-income settings, with limited data from low- and middle-income countries (LMICs). Structural, educational, and systemic disparities in these contexts may influence access to emerging dental technologies, postgraduate training opportunities, and continuing professional development, potentially widening the gap between evidence generation and clinical implementation [10, 11]. Generating context-specific evidence from underrepresented settings is therefore essential to inform strategies that support equitable and evidence-based integration of bioactive materials into clinical practice.

Understanding how dentists perceive and integrate bioactive materials into routine clinical care is critical to identifying knowledge–practice gaps and informing educational and policy interventions. Therefore, this study aimed to compare knowledge, self-perceived understanding, and clinical integration of bioactive dental materials between general dentists and paediatric dentists in a middle-income setting. It was hypothesised that paediatric dentists would demonstrate significantly higher conceptual familiarity and frequency of clinical use compared with general dentists.

## Methods

### Study design and participants

This cross-sectional survey was conducted among licensed dentists practising in Costa Rica in 2025. The study was designed following established reporting recommendations for cross-sectional studies.

According to national registry data, 6029 dentists were actively registered at the time of data collection, including 105 paediatric dentists. Eligible participants were general dentists and paediatric dentists actively engaged in clinical practice with at least six months of professional experience. Dentists from other specialties, residents without formal certification, and those exclusively involved in academic or administrative roles were excluded.

### Sample size and sampling strategy

The minimum sample size was estimated using a precision-based approach, assuming a 95% confidence level, a 5% maximum allowable error, and expected variability values of 21% for general dentists and 12.75% for paediatric dentists. Based on these parameters, the target sample was 233 participants (178 general dentists and 55 paediatric dentists). The original calculation was intended to provide adequate precision for estimating the study parameters and was not based on a prespecified between-group effect size or β error.

A non-probabilistic convenience sampling strategy with voluntary participation was used. A total of 240 complete questionnaires were ultimately included in the analysis, comprising 185 general dentists and 55 paediatric dentists.

### Survey instrument

Data were collected using a structured, self-administered online questionnaire developed and administered through Google Forms. The instrument consisted of 25 items organised into three thematic domains.

The first domain assessed sociodemographic and professional characteristics, including age, gender, years of clinical experience, practice sector (public and/or private), and geographic distribution of practice. The second domain evaluated knowledge related to bioactive dental materials, including familiarity with the concept of bioactivity, recognition of definitions, self-perceived theoretical understanding, and sources of prior training. The third domain explored clinical practice patterns, including frequency of use, categories of bioactive materials employed, and their therapeutic applications in preventive, restorative, and endodontic procedures.

Topical fluoride agents (varnish or gel) were intentionally included as response options to explore potential conceptual overlap in participants’ understanding of bioactivity. Although not classified as bioactive restorative materials under current consensus definitions, their inclusion enabled evaluation of ambiguity in clinical interpretation.

Items assessing knowledge and clinical use were presented using Likert-type scales and multiple-choice formats.

### Instrument validation

Content validity was assessed by three experts in restorative dentistry, biomaterials, and paediatric dentistry, who independently reviewed the questionnaire for relevance, clarity, and consistency with the study objectives. Their feedback resulted in minor wording and terminology adjustments intended to improve the clarity and comprehension of selected items, without modifying the domains assessed or the overall structure of the questionnaire.

The revised instrument was subsequently pilot-tested with 10 dentists who were not included in the final sample. Participants assessed item comprehension, clarity, and completion time. Based on their feedback, additional minor wording adjustments were made to improve readability and response interpretation; no substantive changes to the questionnaire structure or content domains were required.

### Data collection

Data were collected between August and September 2025. Participants were recruited through institutional mailing lists, professional social media groups targeting practising dentists, and in-person outreach at dental clinics and academic events.

The questionnaire remained open for eight weeks, during which periodic reminders were distributed to enhance participation. Digital informed consent was obtained from all participants prior to survey access. Participation was voluntary, anonymous, and uncompensated, and no personally identifiable information was collected.

### Statistical analysis

Data analysis was performed using IBM SPSS Statistics version 22.0 (IBM Corp., Armonk, NY, USA). Descriptive statistics included absolute frequencies, percentages, means and standard deviations, or medians and interquartile ranges, as appropriate. The normality of continuous variables was assessed using the Kolmogorov–Smirnov test prior to selecting parametric or non-parametric tests. Comparisons between professional groups were performed using the Chi-square test or Fisher’s exact test for categorical variables, Student’s t-test for normally distributed continuous variables, and the Mann–Whitney U test for ordinal or non-normally distributed variables.

Multivariable logistic regression analyses were performed to examine whether professional group was independently associated with knowledge and clinical use of bioactive dental materials after adjustment for potential confounding variables. The outcomes examined included correct identification of the definition of a bioactive dental material (correct vs. incorrect), high or very high self-perceived knowledge (vs. moderate, low, or very low), and use of bioactive materials in at least 25% of clinical cases ( ≥ 25% vs. < 25%). Primary models included professional group (general dentist vs. paediatric dentist), age, sex, geographic practice location (Central Valley vs. outside the Central Valley), and public-sector involvement (yes vs. no).

Because age and years of professional experience represent closely related measures of professional seniority, they were not entered simultaneously in the primary models to minimise potential multicollinearity. Sensitivity analyses were performed by replacing age with categories of professional experience.

Adjusted odds ratios (aORs) with 95% confidence intervals (CIs) were reported. All statistical tests were two-tailed, and statistical significance was set at *p* < 0.05. Analyses were conducted using complete-case data; missing data were minimal and were not imputed.

### Ethical considerations

The study protocol was reviewed and approved by the Scientific Ethics Committee of the University of Costa Rica (CEC-UCR; approval code CEC-410-2025). All procedures complied with the ethical principles of the Declaration of Helsinki and applicable national data protection regulations.

Participation was voluntary, anonymous, and uncompensated, and informed consent was obtained electronically prior to questionnaire completion.

## Results

A total of 240 dentists participated, including 185 general dentists (77.1%) and 55 paediatric dentists (22.9%). General dentists had a mean age of 36.5 years (95% CI: 35.4–37.5), compared with 46.0 years (95% CI: 43.0–48.9) among paediatric dentists (*p* = 0.001). Paediatric dentists also had a longer duration of professional practice, with a median of 18 years (IQR: 12–24) compared with 8 years (IQR: 4–14) among general dentists (*p* = 0.003). Most participants were women (79.6%), with no significant difference in sex distribution between groups (*p* = 0.284).

A greater proportion of paediatric dentists had more than 10 years of professional practice compared with general dentists (67.3% vs. 42.2%). Public-sector involvement was reported by 18.3% of participants and did not differ significantly between professional groups (24.0% of paediatric dentists vs. 17.0% of general dentists; *p* = 0.247). Teaching activity was more frequent among paediatric dentists than among general dentists (16.0% vs. 2.0%; *p* = 0.021) (Table 1).

**Table 1:** Sociodemographic and professional characteristics of participating dentists.

Variable	Category	General dentists (*n* = 185)	Paediatric dentists (*n* = 55)	Total	*p*-value
Gender	Female	147 (79.5%)	44 (80.0%)	191 (79.6%)	0.284
	Male	37 (20.0%)	10 (18.2%)	47 (19.6%)	
	Prefer not to respond	1 (0.5%)	1 (1.8%)	2 (0.8%)	
Age (years)	Mean (95% CI)	36.5 (35.4–37.5)	46.0 (43.0–48.9)	38.7 (37.5–39.8)	0.001^a^
Years of professional practice	Median (IQR)	8 (4–14)	18 (12–24)	—	0.003^a^
	0–5 years	56 (30.3%)	7 (12.7%)	63 (26.3%)	
	6–10 years	51 (27.5%)	11 (20.0%)	62 (25.8%)	
	> 10 years	78 (42.2%)	37 (67.3%)	115 (47.9%)	
Public-sector involvement	Yes	31 (17.0%)	13 (24.0%)	44 (18.3%)	0.247
Teaching activity	Yes	4 (2.0%)	9 (16.0%)	13 (5.4%)	0.021^a^

Age is expressed as mean and 95% confidence interval (CI), and years of professional practice as median and interquartile range (IQR). Categorical variables are presented as absolute frequencies (*n*) and percentages (%).

^a^Statistically significant difference between professional groups (*p* < 0.05).

**Fig. 1:**
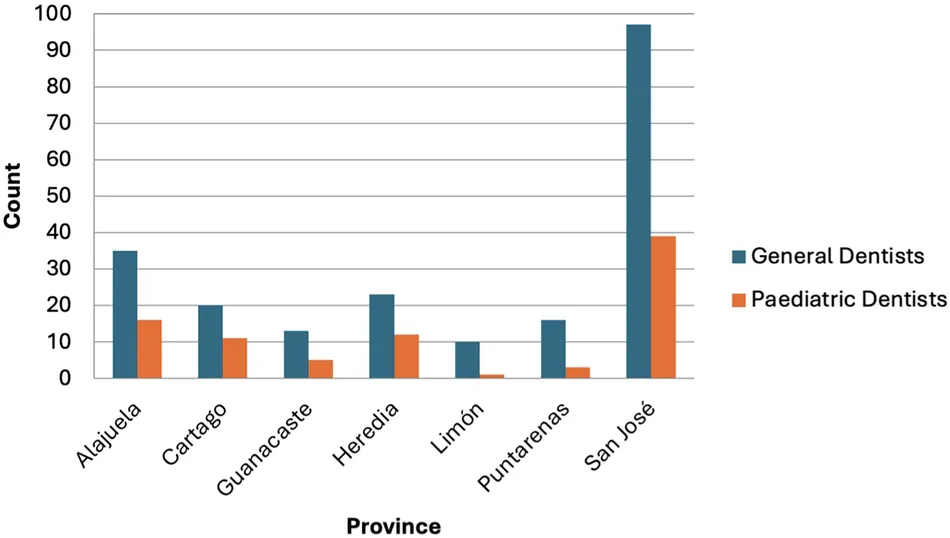
Distribution of participating dentists by province in Costa Rica, stratified by professional group.

Geographical distribution showed a marked concentration of respondents in the Central Valley, particularly in San José, with lower representation from coastal provinces (Fig. 1).

Familiarity with the concept of bioactivity was reported by 84.2% of participants and was significantly higher among paediatric dentists than among general dentists (98.2% vs. 80.0%; *p* = 0.001).

Among participants familiar with bioactive dental materials (*n* = 202), university education was the most frequently reported source of knowledge (61.9%), followed by self-directed scientific reading (35.6%) and professional digital content or networks (32.2%). Courses abroad were reported more frequently by paediatric dentists (33.3%) than by general dentists (6.8%), whereas professional digital content or networks were more frequently reported by general dentists (37.2% vs. 18.5%) (Table 2).

**Table 2:** Knowledge and educational background related to bioactive dental materials by dentist group.

Variable	Category	General dentists *n* (%)	Paediatric dentists *n* (%)	Total *n* (%)	*p*-value
**Familiarity with the term “bioactivity”**	Yes	148 (80.0%)	54 (98.2%)	202 (84.2%)	0.001*
	No	37 (20.0%)	1 (1.8%)	38 (15.8%)	
**Main source of learning**^**a**^	University training	96 (64.9%)	29 (53.7%)	125 (61.9%)	—
	Self-directed study (scientific reading)	50 (33.8%)	22 (40.7%)	72 (35.6%)	—
	Professional digital content / networks	55 (37.2%)	10 (18.5%)	65 (32.2%)	—
	National congress	36 (24.3%)	8 (14.8%)	44 (21.8%)	—
	Course in Costa Rica	17 (11.5%)	16 (29.6%)	33 (16.3%)	—
	Course abroad	10 (6.8%)	18 (33.3%)	28 (13.9%)	—
**Perceived adequacy of undergraduate/postgraduate training**^**b**^	Partially sufficient	62 (41.9%)	17 (31.5%)	79 (39.1%)	0.023*
	Insufficient	50 (33.8%)	12 (22.2%)	62 (30.7%)	
	Term “bioactive” not used during training	19 (12.8%)	15 (27.8%)	34 (16.8%)	
	Fully sufficient	17 (11.5%)	10 (18.5%)	27 (13.4%)	

Values are presented as absolute frequencies (*n*) and percentages (%). Familiarity with the term “bioactivity” was assessed in the full sample (*n* = 240; 185 general dentists and 55 paediatric dentists).

^a^Sources of learning were assessed among participants who reported familiarity with bioactive dental materials (*n* = 202; 148 general dentists and 54 paediatric dentists). Multiple responses were allowed; therefore, totals may exceed 100%.

^b^Perceived adequacy of training was assessed among participants who reported familiarity with bioactive dental materials and provided a response to this item (*n* = 202; 148 general dentists and 54 paediatric dentists). Percentages were calculated within each professional group.

*Statistically significant difference between professional groups (*p* < 0.05; Chi-square test).

Perceived adequacy of academic training differed significantly between professional groups (*p* = 0.023). Overall, 39.1% considered their training partially sufficient, 30.7% considered it insufficient, 16.8% reported that the term “bioactive” was not used during their training, and 13.4% considered their training fully sufficient (Table 2).

Objective knowledge of bioactive dental materials was high overall, with 78.8% correctly identifying the definition. This proportion was higher among paediatric dentists (92.7%) than among general dentists (74.6%). Self-perceived knowledge also differed significantly between groups (χ²(2) = 11.69; *p* = 0.002), with higher levels reported among paediatric dentists and lower levels more frequently reported among general dentists (Table 3).

**Table 3:** Objective and self-perceived knowledge of bioactive dental materials by dentist group.

Type of Knowledge	Indicator / Question	Category	General Dentists ( =*n* 185)	Paediatric Dentists ( =*n* 55)	Total (%)
**Objective Knowledge**	Correct definition of bioactive material	Responded correctly	138 (74.6)	51 (92.7)	78.8
		Responded incorrectly / no answer	47 (25.4)	4 (7.3)	21.2
**Self-Perceived Knowledge**	Self-perceived theoretical and clinical knowledge	Very low / Low	36 (24.3)	5 (9.3)	20.3
		Moderate	79 (53.4)	25 (46.3)	51.5
		High / Very high	33 (22.3)	24 (44.4)	28.2

Values are presented as absolute frequencies (*n*) and percentages (%). Percentages are calculated within each dentist group. A statistically significant difference was observed in self-perceived knowledge levels between groups (χ²(2) = 11.69; *p* = 0.002).

Recognition of bioactive materials varied between professional groups (Table 4). The most frequently identified materials were glass ionomer cement (52.5%), mineral trioxide aggregate (47.9%), and resin-modified calcium silicates (47.5%). Differences between professional groups were most evident for giomers (40.0% vs. 12.4%) and hydroxyapatite/calcium phosphate–based materials (45.5% vs. 28.1%). Only 1.7% of participants were unable to identify any bioactive material.

**Table 4:** Recognition of bioactive dental materials and perceived clinical benefits by specialty.

Variable	Category	General Dentists (*n* = 185), *n* (%)	Paediatric Dentists (*n* = 55), *n* (%)	Total (%)
**Materials identified as bioactive** ^**a**^	Glass ionomer cement	84 (45.4)	42 (76.4)	52.5
	Mineral trioxide aggregate (MTA)	81 (43.8)	34 (61.8)	47.9
	Resin-modified calcium silicates (TheraCal®, UltraBlend®)	85 (45.9)	29 (52.7)	47.5
	Calcium hydroxide	76 (41.1)	28 (50.9)	43.3
	Topical fluoride (varnish or gel)	60 (32.4)	20 (36.4)	33.3
	Pastes or membranes with hydroxyapatite/calcium phosphate	52 (28.1)	25 (45.5)	32.1
	Giomer	23 (12.4)	22 (40.0)	18.8
	Not sure / does not know	3 (1.6)	1 (1.8)	1.7
**Knowledge of clinical benefits**	Yes	119 (64.3)	44 (80.0)	67.9
	No	3 (1.6)	2 (3.6)	2.1
	No response	63 (34.1)	9 (16.4)	30.0
**Recognized benefits** ^**a**^	Promote remineralization	113 (61.1)	43 (78.2)	65.0
	Reduce dentin hypersensitivity	92 (49.7)	42 (76.4)	55.8
	Decrease bacterial acid production	50 (27.0)	23 (41.8)	30.4
	Reduce microleakage at tooth–restoration interface	46 (24.9)	23 (41.8)	28.8
	Modulate biofilm growth	39 (21.1)	16 (29.1)	22.9
	Inhibit biofilm formation	31 (16.8)	14 (25.5)	18.8
	Improve adhesion	31 (16.8)	13 (23.6)	18.3
	Improve physical properties	28 (15.1)	12 (21.8)	16.7
	Improve aesthetics	3 (1.6)	3 (5.5)	2.5

Values are presented as absolute frequencies (*n*) and percentages (%).

^a^Multiple responses were allowed; therefore, totals may exceed 100%. Percentages were calculated based on valid responses for each item (*n* = 163–220). Topical fluoride agents (varnish or gel) were included as response options in the survey instrument.

Regarding perceived clinical effects, 67.9% of participants reported awareness of therapeutic benefits. The most frequently recognised benefits were enamel remineralisation (65.0%) and reduction of dentin hypersensitivity (55.8%), followed by decreased bacterial acid production (30.4%) and reduced microleakage (28.8%). Recognition was generally higher among paediatric dentists (Table 4).

Overall, 65.3% of participants reported using bioactive materials in clinical practice. While the proportion of users did not differ significantly between professional groups, frequency of use was higher among paediatric dentists (χ²(3) = 9.18; *p* = 0.027). General dentists more commonly reported use in less than 25% of cases, whereas paediatric dentists more frequently reported use in at least 25% of cases (Table 5).

**Table 5:** Frequency of bioactive dental material use in clinical practice by dentist group.

Percentage of use in daily practice	General dentists (*n* = 122)	Paediatric dentists (*n* = 46)	Total (*n*, %)
Less than 25% of cases	60 (49.2)	11 (23.9)	71 (42.3)
25–50% of cases	35 (28.7)	18 (39.1)	53 (31.5)
51–75% of cases	21 (17.2)	14 (30.4)	35 (20.8)
More than 75% of cases	6 (4.9)	3 (6.5)	9 (5.4)
**Total**	**122 (100.0)**	**46 (100.0)**	**168 (100.0)**

**Note**. Values are presented as absolute frequencies (*n*) and percentages (%). Percentages were calculated among respondents who reported using bioactive materials in clinical practice (*n* = 168). A statistically significant difference in frequency of use was observed between professional groups (χ²(3) = 9.18; *p* = 0.027).

The most frequently reported clinical objective was restorative treatment (89.0%), followed by preventive applications (72.0%). Management of dentin hypersensitivity (43.0%), endodontic procedures (34.0%), and implantology (8.0%) were less frequently reported, with no significant differences between professional groups (χ²(4) = 4.82; *p* = 0.316) (Table 6).

**Table 6:** Clinical objectives for the use of bioactive dental materials by dentist group.

Clinical objective	General dentists (*n* = 122)	Paediatric dentists (*n* = 46)	Total (%)
Restorative purposes	109 (89.3)	40 (87.0)	89.0
Preventive applications	85 (69.7)	35 (76.1)	72.0
Management of dentin hypersensitivity	60 (49.2)	13 (28.3)	43.0
Endodontic procedures	39 (32.0)	18 (39.1)	34.0
Implantology	13 (10.7)	0 (0.0)	8.0

**Note**. Values are presented as absolute frequencies (*n*) and percentages (%). Percentages were calculated among respondents who reported using bioactive materials in clinical practice (*n* = 168). Multiple responses were allowed; therefore, totals may exceed 100%. No statistically significant differences were observed between groups (χ²(4) = 4.82; *p* = 0.316).

Glass ionomer cements were the most frequently used materials (85.7%), followed by resin-modified calcium silicates (75.0%) and calcium silicate–based cements (63.1%). Calcium hydroxide–based materials were reported by 48.2% of respondents. Less frequently used materials included hydroxyapatite/calcium phosphate–based products (16.7%) and giomers (13.7%). Higher use of giomers was observed among paediatric dentists, although this difference was not statistically significant (*p* = 0.262). Topical fluoride agents were also frequently reported; however, these were included as survey options and are not classified as bioactive restorative materials (Table 7).

**Table 7:** Most commonly used bioactive dental materials in clinical practice by dentist group.

Bioactive Material	General Dentists (*n* = 122), *n* (%)	Paediatric Dentists (*n* = 46), *n* (%)	Total (*n* = 168), *n* (%)
Glass ionomer cements	100 (82.0)	44 (95.7)	144 (85.7)
Calcium silicate–based cements (e.g., mineral trioxide aggregate [MTA], Biodentine®)	76 (62.3)	30 (65.2)	106 (63.1)
Resin-modified calcium silicates (e.g., TheraCal®, UltraBlend®)	98 (80.3)	28 (60.9)	126 (75.0)
Calcium hydroxide–based materials	53 (43.4)	23 (50.0)	81 (48.2)
Hydroxyapatite/calcium phosphate–based products	23 (18.9)	5 (10.9)	28 (16.7)
Giomer-based restorative materials	10 (8.2)	13 (28.3)	23 (13.7)
Topical fluoride agents (varnish or gel)^a^	91 (74.6)	41 (89.1)	132 (78.6)

**Note**. Values are presented as absolute frequencies (*n*) and percentages (%). Percentages were calculated among respondents who reported using the listed materials (*n* = 168). Multiple responses were allowed; therefore, totals may exceed 100%.

^a^Topical fluoride agents were included as survey response options but are not classified as bioactive restorative materials according to current definitions.

Across both groups, 68.5% of respondents agreed that the use of bioactive materials had a positive impact on clinical outcomes.

In multivariable analyses, paediatric dentists had significantly higher odds than general dentists of correctly identifying the definition of a bioactive dental material after adjustment for age, sex, geographic practice location, and public-sector involvement (aOR = 4.98; 95% CI: 1.57–15.74; *p* = 0.006). Paediatric dentists also had higher odds of reporting high or very high self-perceived knowledge (aOR = 2.60; 95% CI: 1.21–5.58; *p* = 0.014) and of using bioactive materials in at least 25% of clinical cases (aOR = 3.26; 95% CI: 1.38–7.69; *p* = 0.007). Age, sex, geographic practice location, and public-sector involvement were not independently associated with these outcomes. In a sensitivity analysis replacing age with years of professional experience, the association between paediatric dentistry and correct conceptual knowledge remained significant (aOR = 4.48; 95% CI: 1.51–13.33; *p* = 0.007).

## Discussion

This study provides a structured assessment of knowledge, conceptual understanding, and clinical integration of bioactive dental materials in a middle-income setting. The findings reveal differences between general and paediatric dentists, together with geographic and educational factors potentially relevant to the incorporation of bioactive technologies into routine practice.

Although experimental and clinical evidence supporting bioactive materials continues to expand, population-level data on how clinicians conceptualise and implement these materials remain limited, particularly in low- and middle-income contexts [2, 4, 12, 13]. By incorporating objective conceptual assessment alongside clinical and contextual variables, this study extends previous survey-based research beyond material preference toward a more comprehensive evaluation of clinical reasoning and definitional clarity.

Overall familiarity with the concept of bioactivity was high; however, important differences emerged in conceptual precision, objective knowledge, and clinical confidence. Paediatric dentists demonstrated greater accuracy in defining bioactivity, higher self-perceived competence, and broader recognition of advanced materials. These findings align with the clinical profile of paediatric dentistry, where ion-releasing and biologically active materials are routinely used in vital pulp therapy and minimally invasive approaches [14–16]. Similar differences between general dentists and paediatric dentists have been reported internationally, suggesting that postgraduate training and repeated clinical exposure may contribute to greater conceptual understanding and sustained adoption of bioactive materials [17–19].

Importantly, these differences persisted in the multivariable analyses after adjustment for demographic and practice-related factors. Paediatric dentists had approximately five-fold higher adjusted odds of correctly identifying the definition of a bioactive material and more than three-fold higher adjusted odds of using bioactive materials in at least 25% of clinical cases compared with general dentists. The associations remained significant after adjustment for age, sex, geographic practice location, and public-sector involvement. Furthermore, sensitivity analyses replacing age with years of professional experience yielded similar estimates, suggesting that the observed differences were not solely attributable to the greater professional seniority of paediatric dentists. Nevertheless, given the cross-sectional design, these findings should not be interpreted as demonstrating that postgraduate specialty training itself caused the observed differences.

The geographic concentration of respondents in urban regions reflects previously documented inequalities in the distribution of dental professionals [11]. Such disparities may indirectly affect exposure to innovation by limiting access to postgraduate programmes, academic networks, and continuing professional development. Comparable structural barriers influencing the diffusion of clinical advances have been described in other settings [10], reinforcing that adoption of emerging biomaterials may be shaped not only by individual knowledge but also by systemic and infrastructural factors.

Educational background also emerged as a potentially relevant factor. Although university training was the main reported source of knowledge, general dentists reported greater reliance on professional digital content, whereas self-directed scientific reading was reported by substantial proportions of both groups. This pattern is consistent with evidence indicating that undergraduate curricula may prioritise operative techniques over biological mechanisms and material–tissue interactions [20]. The substantial proportion of respondents who perceived their training as partially sufficient or insufficient suggests a potential need to strengthen the integration of bioactivity concepts within undergraduate dental education [21, 22].

Recognition of bioactive materials varied across categories. Well-established materials, such as glass ionomer cements and mineral trioxide aggregate, were widely recognised, reflecting their long-standing clinical use and inclusion in dental curricula. In contrast, giomers and hydroxyapatite/calcium phosphate–based materials were less frequently identified, particularly among general dentists. Previous studies indicate that familiarity is influenced by clinical longevity, guideline inclusion, and market availability, while newer materials may face barriers related to cost and uncertainty regarding long-term performance [4, 23].

Notably, although recognition of giomers was higher among paediatric dentists, their reported clinical use remained limited. This discrepancy highlights a knowledge–practice gap between conceptual awareness and routine implementation. Similar patterns have been described in the adoption of emerging dental technologies, where economic factors, material availability, and uncertainty regarding long-term outcomes influence clinical uptake [24]. In this context, preference for more established calcium silicate–based materials may also contribute to selective adoption.

Approximately two-thirds of respondents reported using bioactive materials in clinical practice, indicating moderate integration. However, frequency of use differed between general and paediatric dentists, with paediatric dentists reporting greater incorporation into clinical practice. This difference persisted in the adjusted analysis, in which paediatric dentists had more than three-fold higher odds of using bioactive materials in at least 25% of clinical cases. This finding is consistent with comparative studies of caries management and regenerative procedures, where clinical philosophy, training, and patterns of patient care may influence material selection [25–27].

The predominance of glass ionomer cements and resin-modified calcium silicates suggests that adoption remains centred on accessible, ion-releasing materials supported by established clinical protocols. Importantly, topical fluoride agents were frequently classified as “bioactive.” However, current definitions require material-driven ion exchange, interfacial interaction, and apatite formation originating from the material itself, criteria not met by topical fluoride agents [1, 2]. This finding highlights a conceptual overlap between preventive fluoride strategies and true material-driven bioactivity, reflecting a persistent area of ambiguity in clinical understanding [9].

Taken together, these findings underscore the need to strengthen biologically oriented restorative frameworks across dental education systems. Integrating structured content on material–tissue interaction and evidence-based decision-making into undergraduate and postgraduate curricula may improve conceptual clarity and clinical application [20, 21]. Expanding continuing professional development, particularly through accessible digital formats, may help reduce disparities in access to knowledge and support more consistent integration of bioactive principles into practice [28, 29].

In Costa Rica, the National Higher Education Accreditation System (SINAES) provides the national framework for quality assurance in higher education, including dental programmes. Its accreditation criteria emphasise continuous quality improvement, curricular updating, incorporation of technologies into professional training, and qualified academic staff, within a framework intended to remain consistent with internationally recognised standards [30]. These principles are broadly aligned with international quality-assurance frameworks in health professions education, which emphasise continuous programme improvement, competency-based education, curricular responsiveness, and the adoption of internationally recognised standards while addressing local and global health needs [31]. However, these broad accreditation frameworks do not specifically prescribe competencies related to emerging dental biomaterials. In this context, the present findings suggest an opportunity to strengthen the explicit integration of contemporary biomaterials science, material–tissue interactions, and evidence-based evaluation of emerging materials within dental curricula.

Beyond education, alignment with consensus-based definitions and clinical guidelines may facilitate a shared understanding of bioactivity and promote more consistent, evidence-based restorative decision-making across different practice settings.

This study has several limitations. Data were self-reported and may not accurately reflect actual clinical behaviour. The voluntary, non-probabilistic sampling strategy represents an important limitation. Recruitment through institutional mailing lists, professional social media groups, academic events, and direct outreach may have preferentially attracted dentists with greater interest in bioactive materials, continuing professional development, or academic activities, resulting in self-selection bias. Consequently, participants may have had greater familiarity with emerging dental materials than non-participants, potentially leading to overestimation of knowledge and clinical adoption. In addition, the geographic concentration of respondents in the Central Valley limits extrapolation to dentists practising in rural and coastal regions, where access to postgraduate education, continuing professional development, and newer dental materials may differ. Therefore, the findings should be interpreted as describing the responding sample rather than as nationally representative estimates. Although multivariable analyses were used to account for measured demographic and practice-related factors, residual confounding from unmeasured variables cannot be excluded.

## Conclusion

Dentists demonstrate high overall awareness of bioactive dental materials; however, substantial variability persists in conceptual understanding and frequency of clinical use. Differences between general and paediatric dentists suggest that postgraduate training and clinical exposure may contribute to the adoption of biologically driven restorative approaches.

Despite this, integration into general dental practice remains uneven, reflecting a knowledge–practice gap that may extend to similar middle-income contexts. Strengthening undergraduate and continuing education, addressing disparities in access to training, and reinforcing consensus-based definitions of bioactivity may support more consistent and evidence-based clinical implementation.

Future research should evaluate how improved conceptual understanding translates into sustained adoption and measurable patient-centred outcomes across diverse practice settings.

## Data Availability

The datasets generated and/or analysed during the current study are available from the corresponding author on reasonable request.
